# Clinical use of ozone therapy: a critical appraisal of evidence, safety, and methodological quality

**DOI:** 10.3389/fmed.2026.1893156

**Published:** 2026-09-03

**Authors:** Francesco Maria Galassi, Enrico Zauli, Mauro Vaccarezza, Elena Varotto

**Affiliations:** 1 Department of Anthropology, Faculty of Biology and Environmental Protection, University of Łódz, Łódz, Poland; 2 Department of Translational Medicine, University of Ferrara, Ferrara, Italy; 3 Curtin School of Diagnostic and Therapeutic Sciences, Curtin University, Bentley, Perth, WA, Australia; 4 Curtin Medical Research Institute (Curtin-MRI), Bentley, Perth, WA, Australia; 5 Department of Environmental and Prevention Sciences, University of Ferrara, Ferrara, Italy; 6 College of Human Sciences and Culture, Flinders University, Adelaide, SA, Australia

**Keywords:** complementary and alternative medicine, evidence-based medicine, medical misinformation, oxidative stress, ozone therapy, regulatory policy

## 1 Introduction

Ozone therapy is frequently promoted as a versatile and minimally invasive medical intervention purported to exert a broad range of biological effects, including anti-inflammatory, antimicrobial, immunomodulatory, and regenerative actions. These claims have led to its application across diverse clinical domains, such as musculoskeletal disorders, dermatological conditions, chronic wounds, peripheral vascular disease, chronic inflammatory diseases, infectious pathologies, and, more recently, viral illnesses. In many private clinical settings, ozone therapy is presented as an adjunct or alternative to established treatments, sometimes accompanied by claims of efficacy that overstate the strength of the underlying evidence. Such representations stand in contrast to the principles of evidence-based medicine, which require therapeutic claims to be supported by reproducible, high-quality clinical evidence. At the same time, as detailed below, ozone therapy is legally recognized as a complementary or traditional medical procedure in a substantial number of jurisdictions, and a growing, heterogeneous body of systematic reviews and meta-analyses—several published in the last 5 years—reports statistically significant benefit for specific indications, alongside other syntheses reporting very-low-certainty or unsupportive evidence for others. This coexistence of supportive, inconclusive, and negative evidence has contributed to ongoing scientific and regulatory debate regarding the appropriate role of ozone therapy in contemporary clinical practice, highlighting the need for balanced evidence synthesis that distinguishes between individual indications rather than evaluating the therapy as a single entity. The present narrative critical review aims to reflect this heterogeneity rather than to characterize ozone therapy uniformly as either validated or disproven, while examining regulatory positions, the quality and direction of clinical studies across indications, historical patterns of adoption, economic and communicative factors, and reported adverse events.

## 2 Review approach and source selection

This article is conceived as a narrative critical review, not a systematic review with a pre-registered protocol, quantitative meta-analysis, or formal risk-of-bias synthesis performed by the present authors. In line with recommended standards for the reporting of narrative reviews (1), we clarify here the approach used to identify and select the literature discussed below. PubMed/MEDLINE, Google Scholar, and the Cochrane Library were searched using combinations of the terms “ozone therapy,” “oxygen-ozone,” “autohemotherapy,” “ozonated,” together with indication-specific terms (e.g., “low back pain,” “disc herniation,” “diabetic foot ulcer,” “peripheral arterial disease,” “osteoarthritis,” “dental caries,” “dermatology”) and methodological filters (“systematic review,” “meta-analysis,” “randomized controlled trial,” “umbrella review”). Regulatory and consensus documents, including national and international ozone-therapy society declarations, were also consulted to characterize the legal and institutional status of the practice. Priority was given to systematic reviews, meta-analyses, and umbrella reviews over individual small trials or case reports, and to publications from 2010 onward, supplemented by earlier landmark meta-analyses (e.g., references 5, 6) where they remain the most complete synthesis available for a given indication. Rather than adjudicating primary studies directly, we relied on the risk-of-bias, GRADE, and AMSTAR-2 judgments reported within these secondary sources. This approach, while allowing broader synthesis across ozone's multiple administration modalities (systemic autohemotherapy, intradiscal or periarticular injection, topical application, rectal insufflation, and gas bathing), does not eliminate selection subjectivity inherent to narrative reviews, and no formal database search log, PRISMA flow diagram, or explicit inclusion/exclusion criteria were applied; this is acknowledged here as a methodological limitation of the present work.

## 3 Regulatory and biological considerations

From a regulatory perspective, ozone occupies a problematic position. The United States Food and Drug Administration (FDA) explicitly classifies ozone as a toxic gas with no proven medical utility in specific, adjunctive, or preventive therapy, emphasizing that concentrations required to achieve germicidal effects are incompatible with human safety, given ozone's strong oxidative properties and its capacity to damage cellular membranes, proteins, and lipids. In marked contrast, ozone therapy is legally recognized as a complementary or traditional medical procedure in a number of countries and jurisdictions, including Greece (regulated since 1991), Ukraine (since 2001), Italy, Spain, Portugal, Turkey, Russia, Germany, China, Cuba, Mexico, Brazil, and the United Arab Emirates, among others, as documented in the consensus Madrid Declaration on Ozone Therapy issued by the International Scientific Committee of Ozone Therapy (ISCO3) (2). The purposes for which it is authorized vary by jurisdiction: in Cuba, ozone therapy is institutionally embedded within the national Natural and Traditional Medicine system and is administered in hospital-based centers, most prominently for diabetic foot ulcers, peripheral vascular insufficiency, and chronic inflammatory conditions, under standardized dosing and contraindication protocols; in several European countries, regulation has instead proceeded at the regional or professional-association level, permitting use by licensed physicians as a complementary adjunct rather than as a first-line therapy. In parts of the United States, ozone therapy may be practiced under general health-freedom or non-allopathic-medicine statutes rather than under a specific therapeutic indication. This heterogeneity illustrates that legal permission reflects jurisdiction-specific regulatory philosophy and does not, by itself, constitute a determination of clinical efficacy. Biologically, ozone is a highly reactive oxidant. While proponents often invoke hormetic or indirect antioxidant mechanisms, such effects remain incompletely substantiated. No fully validated pharmacokinetic or dose–response model yet reconciles ozone's oxidative reactivity with a consistent, generalisable therapeutic benefit across the heterogeneous modalities in which it is applied, although, as discussed below, mechanistic and clinical evidence for specific effects (e.g., growth-factor induction in wound healing, rheological changes in ischaemic tissue) has accumulated for particular indications.

## 4 Clinical evidence and methodological quality

Ozone therapy is not a single intervention but a heterogeneous family of modalities—systemic autohemotherapy, intradiscal or periforaminal injection, topical application, rectal insufflation, and gas bathing—applied across markedly different clinical contexts. The level and direction of the available evidence differ substantially by modality and indication, and general conclusions about “ozone therapy” as an undifferentiated category risk obscuring these differences.

For several indications, systematic reviews report low or very low certainty evidence that does not support a clinical recommendation. In dentistry, a systematic review and meta-analysis concluded that the available evidence for ozone in the treatment of dental caries is of very low certainty (3). In dermatology, a comprehensive review reported that studies addressing acne, ulcerations, dermatitis, and herpes are largely preliminary and methodologically inconsistent, without standardized protocols (4). In musculoskeletal medicine, an umbrella review focusing on knee osteoarthritis found that all included systematic reviews were rated as critically low quality according to the AMSTAR-2 tool (5).

For other indications, however, systematic reviews and meta-analyses—several from the last 5 years—report statistically significant benefit, albeit with important caveats. For chronic low back pain due to lumbar disc herniation, an early systematic review and meta-analysis of randomized controlled trials concluded that percutaneous ozone injection yielded positive results and low morbidity, while cautioning that this conclusion rested on a small number of studies with methodological limitations and possible publication bias (6). A larger meta-analysis of 12 studies (approximately 8,000 patients) reported mean improvements in pain and Oswestry Disability Index scores comparable to those achieved with surgical discectomy, alongside a low complication rate (0.064%) (7). A more recent systematic review and meta-analysis found that ultrasound-guided periforaminal ozone infiltration produced treatment success rates at six months that were superior to those of transforaminal epidural steroid injection (8). These studies are nonetheless predominantly small, frequently non-blinded or quasi-experimental, and heterogeneous in ozone concentration, injection technique, and comparator, which limits the strength and generalisability of the conclusions.

For chronic wounds, a systematic review of seven studies (506 patients, mostly with diabetic foot ulcers) found a consistent positive healing effect of topical ozone with no reported adverse events, while noting that the evidence derived largely from non-randomized designs and calling for further randomized controlled trials (9). A systematic review restricted to randomized controlled trials similarly found that ozone significantly improved wound area and lowered amputation rates specifically for diabetic foot ulcers, while noting that evidence for other refractory wound types remained insufficient for meta-analysis (10). A 2024 meta-analysis of 11 studies (960 patients) found that ozone therapy significantly reduced ulcer size, shortened healing time, decreased hospital length of stay, and reduced amputation rates compared with standard treatment, while explicitly noting that its effect did not differ from standard treatment for achieving complete ulcer resolution—a nuanced finding indicating partial rather than unequivocal benefit (11).

For peripheral arterial disease and critical limb ischaemia, a narrative review of *in vitro*, animal, and clinical studies concluded that oxygen–ozone therapy improves tissue perfusion, glycaemic control, and rheology, reduces amputation risk in diabetic foot complications, and may lower treatment costs by approximately one quarter compared with standard antibiotic therapy, with no adverse events reported across the included clinical trials; the authors non-etheless emphasized that the underlying clinical evidence derives predominantly from small, non-randomized studies lacking long-term outcome data, and called explicitly for larger controlled trials (12).

Taken together, recurring methodological limitations across both the negative and the positive syntheses include small sample sizes, inadequate randomization and blinding (difficult to achieve given the gas's detectable odor and the procedural nature of most interventions), heterogeneous outcome measures, and the possibility of publication bias favoring positive results. Importantly, the current literature more often reflects an absence of high-certainty evidence than definitive proof of either efficacy or inefficacy—a distinction that is central to interpreting clinical uncertainty within an evidence-based framework, and one that argues against treating ozone therapy as a monolithic, uniformly disproven, or uniformly validated intervention.

## 5 Historical perspective

Ozone was isolated in 1840 by Christian Friedrich Schönbein, who first described its characteristic pungent odor and recognized its strong oxidative properties. In the late nineteenth and early twentieth centuries, ozone attracted intermittent medical interest, primarily due to its presumed disinfectant potential. During the first half of the twentieth century, particularly in military and emergency medical contexts, ozone was empirically applied to the treatment of infected wounds and ulcers, at a time when antibiotics were either unavailable or in limited supply. The widespread introduction of penicillin and subsequent generations of antibiotics during the mid-twentieth century substantially reduced clinical interest in ozone as an antimicrobial treatment, as pharmacological therapies offered more predictable efficacy, standardized dosing, and a rapidly expanding evidence base. These early applications preceded the development of modern clinical trial methodology and were conducted without standardized protocols, control groups, or systematic assessment of efficacy and safety. Unlike medical technologies such as hyperbaric oxygen therapy, dialysis, or extracorporeal membrane oxygenation—which emerged from experimental research and subsequently underwent rigorous clinical validation, regulatory scrutiny, and institutional adoption—ozone therapy has followed a markedly more fragmented trajectory, achieving formal institutional integration in some health systems (e.g., Cuba, as noted above) while remaining largely outside academic medicine and university-based training in others, including the United States. The consolidation of evidence-based medicine during the 1990's reinforced the requirement that therapeutic interventions demonstrate efficacy through well-designed randomized controlled trials and systematic evidence synthesis; within this evolving framework, ozone therapy's continued reliance on private-sector training programmes, specialized courses, and dedicated international associations, operating substantially outside conventional regulatory and educational structures, has limited its broader integration even where isolated indications now have supportive systematic evidence.

## 6 Linguistic and cognitive bias

In addition to biomedical considerations, several contextual factors discussed in the health-communication and risk-perception literature may contribute to the persistence of certain medical practices independent of the strength of the underlying clinical evidence. A well-documented phenomenon in judgment and decision-making research is the affect heuristic, whereby individuals substitute an immediate emotional impression for a more effortful analytic assessment of risk and benefit, particularly when information is incomplete, technical, or ambiguous; experimental work has repeatedly shown an inverse relationship between perceived benefit and perceived risk that tracks the valence of the underlying affective association rather than objective probability (13). Within health communication research specifically, the framing and affective tone of the language used to describe a condition, or intervention has been shown to shape patients' risk perceptions and treatment preferences (14). An often-overlooked contributor to the appeal of ozone therapy is linguistic association: in public discourse, the term “ozone” is strongly linked to the atmospheric ozone layer, commonly portrayed as a protective shield against harmful ultraviolet radiation. While no study has, to our knowledge, directly tested this specific semantic association for the word “ozone,” the broader affect-heuristic literature provides a plausible psychological mechanism by which such positive framing could foster an intuitive perception of the therapy as inherently protective, independent of clinical evidence, and could accordingly shape patient expectations and public understanding of the intervention (13, 14).

## 7 Economic drivers

Commercial market-research reports estimate that the global market for ozone therapy devices and related services was valued at approximately USD 579.7 million in 2024 and project growth to approximately USD 980.9 million by 2034, at a compound annual growth rate of roughly 5.4%, driven largely by expanding use in wound care, pain management, and dermatology (15). These figures derive from proprietary commercial market-research methodology rather than peer-reviewed health-economics analysis, and, to our knowledge, no rigorous cost-effectiveness evaluation comparing ozone therapy with standard care has been published for most indications; the diabetic-foot cost data noted in the peripheral arterial disease literature above, suggesting savings of roughly one quarter relative to standard antibiotic therapy, remain among the few empirical exceptions (12). One structural factor sometimes proposed to explain the persistence of a commercial market for a non-patentable natural gas is the absence of exclusive intellectual-property protection, which limits the financial incentive for large pharmaceutical sponsors to fund the large multicentre randomized trials that formal regulatory approval typically requires; this explanation has been advanced within the ozone-therapy professional literature itself (2); however, to the best of our knowledge, it has not been systematically investigated or empirically validated through dedicated health-economics, market-analysis, or medical-sociology studies. Accordingly, we present this explanation as a plausible hypothesis that may contribute to understanding the therapy's continued commercial presence rather than as an evidence-based determinant of its market persistence. This distinction is important because direct empirical evidence specifically addressing this proposed mechanism is currently lacking.

## 8 Risks and documented adverse events

Contrary to frequent claims of safety and minimal invasiveness, ozone therapy is associated with a range of documented risks, though these appear concentrated in specific administration routes rather than distributed evenly across all modalities. Severe complications, including fatal gas embolism, have been reported following ozonated autohemotherapy,underscoring the inherent dangers of introducing gas mixtures into the vascular system (16). In addition, a well-documented outbreak of hepatitis C virus infection linked to ozone-enriched autologous blood transfusion highlights the risks posed by non-standardized procedures and inadequate infection control in systemic, blood-handling applications (17). By contrast, the localized, topical, and intradiscal modalities reviewed in Section 4 above did not report comparable adverse events in the cited systematic reviews, although the absence of standardized, mandatory adverse-event reporting for ozone therapy overall means that the true incidence of complications, across all modalities, remains difficult to quantify with confidence. This asymmetry reinforces the importance of evaluating procedural safety by administration route rather than treating ozone therapy as a single risk category. It also underscores the need for standardized treatment protocols, rigorous operator training, and systematic adverse-event surveillance to enable more reliable assessment of the therapy's overall safety profile.

## 9 Ethical and practical considerations

Patients should be fully informed of the current, heterogeneous state of the evidence and of the documented risks associated with specific administration routes. The disclosure obligation inherent in informed consent does not vary according to whether an intervention is labeled standard, experimental, complementary, or traditional: physicians and other qualified providers bear a responsibility to communicate the best available evidence on an intervention's therapeutic worth regardless of its regulatory or disciplinary label, and misrepresentation of material facts about therapeutic worth can void consent and carry legal consequences (18). Applied to ozone therapy, this principle means clearly conveying to patients both the indications for which recent systematic evidence suggests plausible, modest benefit—for example, chronic radicular low back pain or diabetic foot ulcers—and the indications for which the evidence remains very low in certainty or unsupportive, such as dental and most dermatological uses, rather than presenting the therapy in uniformly positive or uniformly dismissive terms. In contexts where clinical efficacy remains uncertain,obtaining truly informed consent becomes particularly important, as patients must be made aware not only of potential risks but also of the modality-specific and indication-specific limits of validated therapeutic benefit. In such situations, shared decision-making assumes particular importance, enabling patients' values, preferences, and therapeutic goals to be considered alongside the best available scientific evidence. Another ethical concern involves the risk of therapeutic misconception, whereby patients may interpret the offer of a medical procedure—particularly when framed using positively valenced terminology such as “ozone” (see Section 6)—as implicit evidence of its effectiveness. Clinicians therefore have a professional responsibility to ensure that communication with patients and the public accurately reflects the current, heterogeneous state of scientific knowledge and does not inadvertently legitimize non-validated applications through the authority of the medical encounter.

## 10 Conclusions

Ozone therapy comprises a heterogeneous family of modalities for which the level and direction of clinical evidence differ substantially by indication and route of administration. For chronic radicular low back pain due to disc herniation, chronic wounds including diabetic foot ulcers, and peripheral arterial disease, recent systematic reviews and meta-analyses report statistically significant, generally modest, benefit, most often graded as low-to-moderate certainty evidence and frequently limited by small, non-randomized, or otherwise methodologically constrained primary studies. For dental caries, dermatological conditions, and knee osteoarthritis, systematic and umbrella reviews report very-low-certainty or critically low-quality evidence that does not currently support a clinical recommendation. Systemic autohemotherapy, in particular, has been associated with rare but severe adverse events, including fatal gas embolism and bloodborne infection transmission, that appear specific to that administration route. We are not undermining promising data from recent literature, nor new methods to improve ozone delivery (19–21), but the emerging consensus across the reviewed indications is that more high-quality clinical data and more randomized controlled trials are needed before firmer conclusions can be drawn (19, 20), and that “much work still needs to be undertaken and new avenues are to be explored” (21). Physicians committed to evidence-based medicine should therefore neither present ozone therapy as a uniformly validated treatment option nor dismiss it as uniformly disproven; rather, they should communicate, indication by indication and modality by modality, the current state and limitations of the evidence, consistent with the ethical principle of providing care grounded in the best available research.
